# Supplementary material for: Templated bilayer self-assembly of fully conjugated π-expanded macrocyclic oligothiophenes complexed with fullerenes
Source: Nat Commun. 2017 Mar 10;8:14717. doi: 10.1038/ncomms14717 (PMC5353715; doi:10.1038/ncomms14717)
Supplement: Supplementary Information — Supplementary Figures, Supplementary Table, Supplementary Notes, Supplementary Methods and Supplementary References [file ncomms14717-s1.pdf]

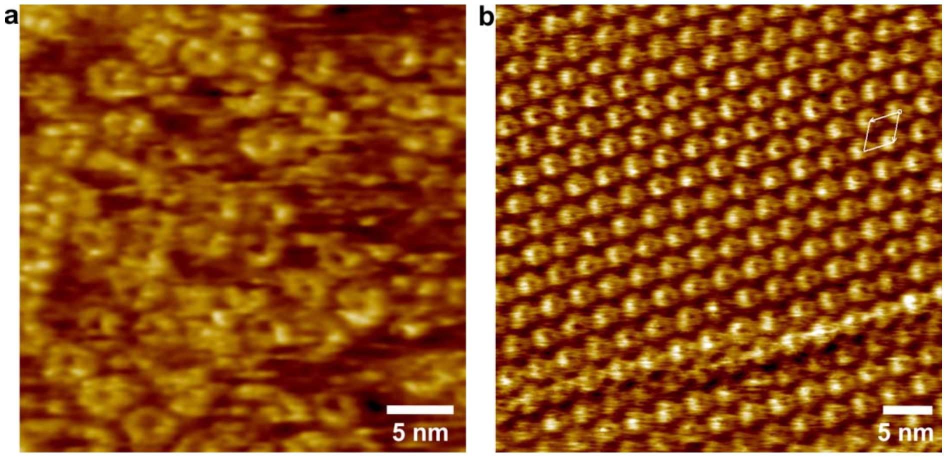

Supplementary Figure 1 | Irregular arrangement of *E,E*-8-mer on TMA. STM height images formed when a 5 µL heptanoic acid solution of *E,E*-8-mer is applied on: (**a**) a TMA templated HOPG substrate, U_s = +1.00 V, I_t = 50 pA; (**b**) HOPG, U_s = -1.12 V and I_t = 58 pA. Unit cell: a = (2.93 ± 0.06) nm, b = (2.95 ± 0.07) nm, θ = (60 ± 3) °.

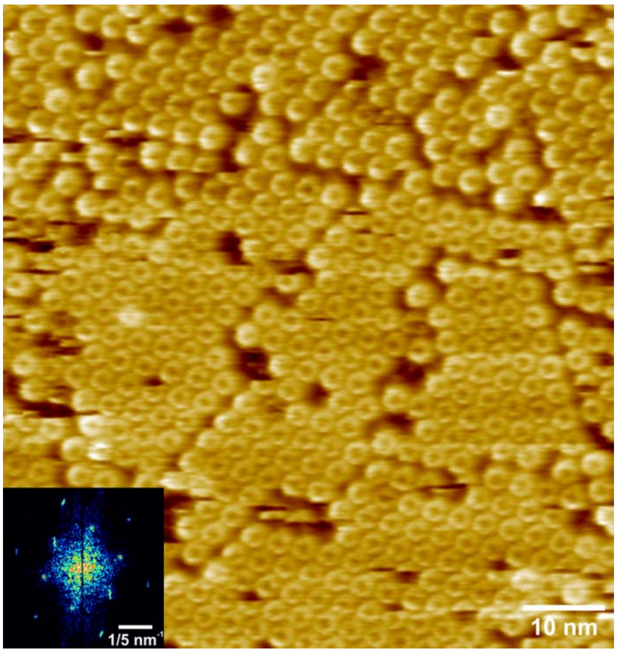

Supplementary Figure 2 | Long-range STM height image of *E,E*-8-mer·C_{60} on TMA. Tunneling conditions: U_s = +1.05 V and I_t = 56 pA. Two unit cells can be determined from the fast Fourier transform (FFT), shown as inset, both corresponding to hexagonal packing, with the axes rotated by 30 °. The smaller one with two complexes at opposite corners: a_1 = (1.69 ± 0.08) nm, b_1 = (1.70 ± 0.07) nm and θ_1 = (61 ± 2) °; and a bigger one with four complexes at the four corners: a_2 = (3.0 ± 0.1) nm, b_2 = (3.0 ± 0.2) nm and θ_2 = (60 ± 1) °.

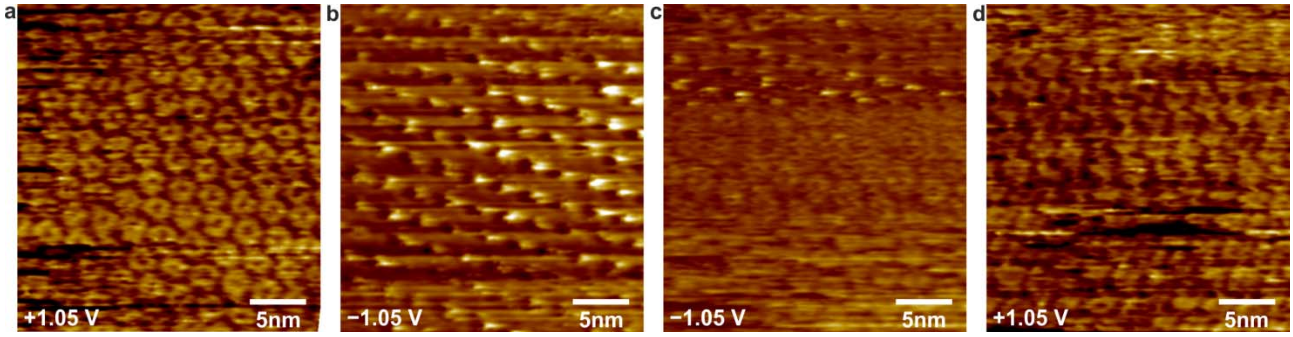

Supplementary Figure 3 | Bias dependent imaging of a templated bilayer of *E,E*-8-mer·C_{60}. STM height images showing a progression of the bias set-point imaging of a templated bilayer of *E,E*-8-mer·C_{60}. Scan time of each image is approximately one minute. Bright spots assigned to C_{60} (**b**) are not visible in the following image (**c**). Switching back to positive substrate bias (**d**), restore the arrangement of moieties as in (**a**).

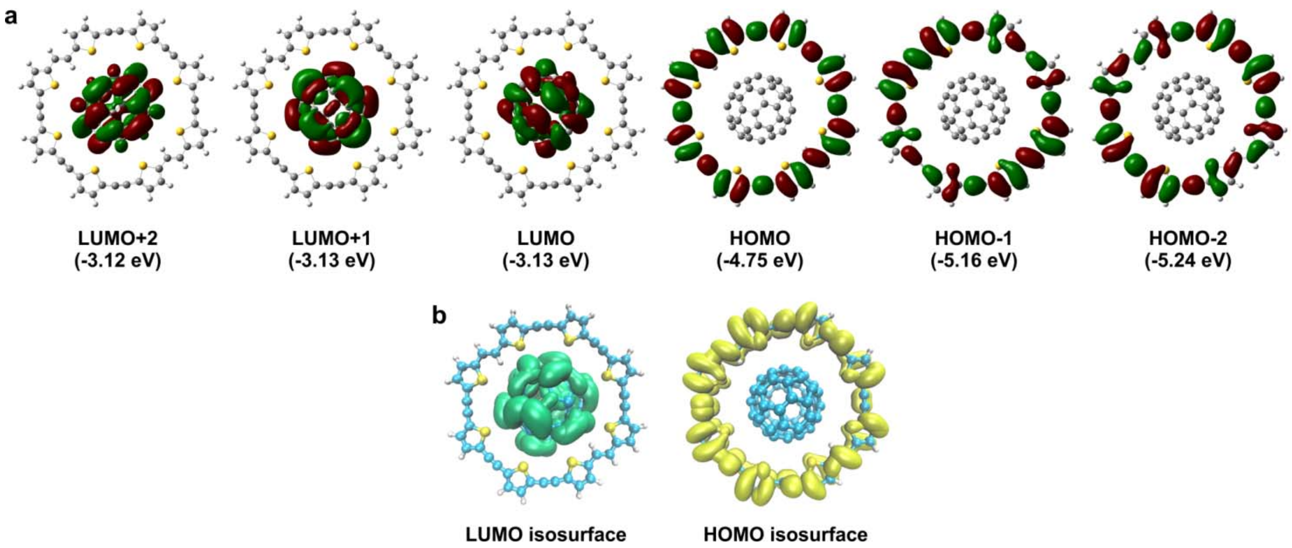

Supplementary Figure 4 | Density Functional Theory molecular orbitals of *E,E*-8-mer·C_{60}. (**a**) Calculated frontier molecular orbitals. (**b**) Isosurfaces showing the electron probability distribution 1 eV below and above the HOMO and LUMO respectively.

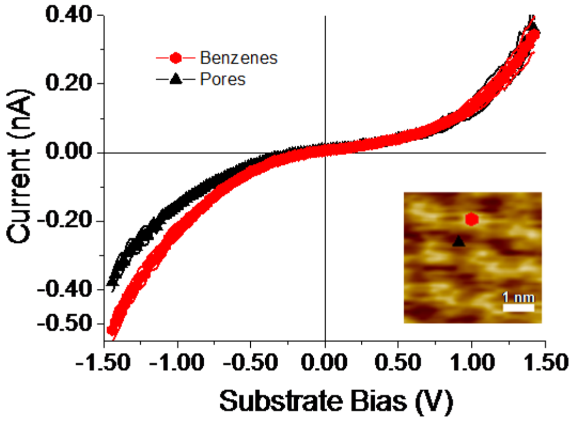

Supplementary Figure 5 | STS measurements on TMA honeycomb-like network. STS on the bright spots (TMA molecules) and in the pores. The curves are lognormal averaged for measurements taken at the bright spot of the image inset (red curve), which we attributed to higher tunneling regions of the sample, named the aromatic cores of the TMA[1], while the black curve is taken on the dark spot of the image, which corresponds to the pores. The current response to opposite biases is symmetric in the case of the black curve, because the transport occurs direct to the HOPG substrate and/or through the solvent molecules, while in the case of the benzene core of the TMA, the current tends to be higher in absolute values at negative bias. In the scope of electronic transport through an asymmetric gap, this means that the higher occupied molecular orbital (HOMO) of the TMA lies closer to the Fermi-level of HOPG than the lowest occupied molecular orbital (LUMO)[2]. Straight lines connect the limits of the error bars for each averaged curve. (STS conditions U_s = −0.86 V and I_t = 100 pA).

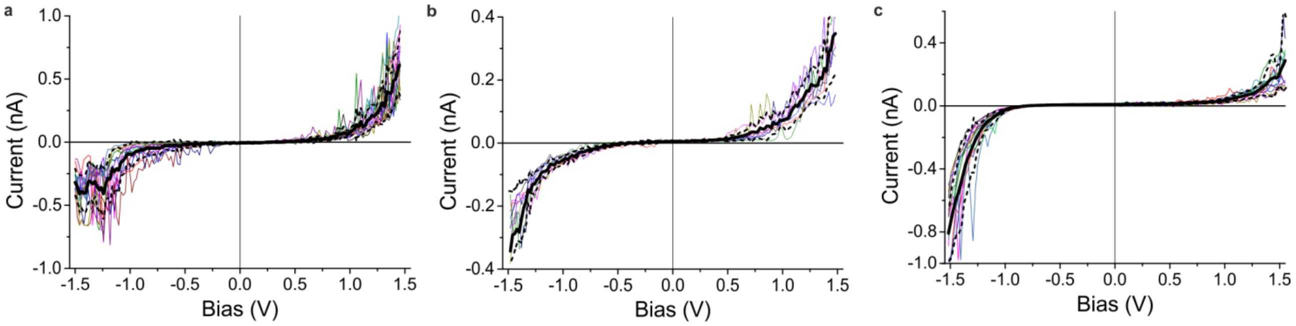

Supplementary Figure 6 | Scattering of averaged I-Vs in Fig. 3. (**a**) 13 different I-V curves through the rim of the complex *E,E*-8-mer·C_{60} (color lines) and the average value (thick black line). (**b**) 8 different I-V curves through the center of the complex *E,E*-8-mer·C_{60} (color lines) and the average value (thick black line). (**c**) 12 different I-V curves through a monolayer of the macrocycle *E,E*-8-mer on HOPG (color lines) and the average value (thick black line). A dashed line connects the limits of the error bars for each graph.

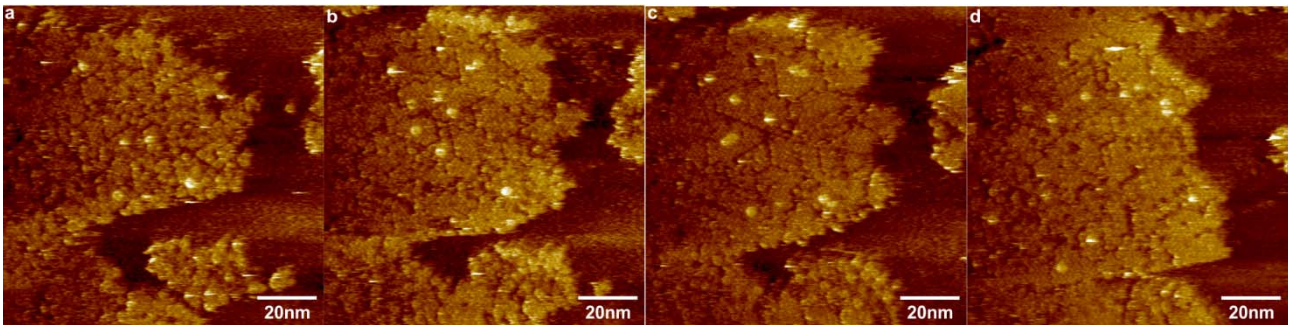

Supplementary Figure 7 | Time evolution of the coverage of *E,E*-8-mer·C_{60} on TMA. STM height images showing the progression of the coverage with approximately two minutes between each figure. U_s = +0.91 V and I_t =65 pA. The lower part of the image is HOPG covered with TMA; after some minutes the islands reached a maximum size.

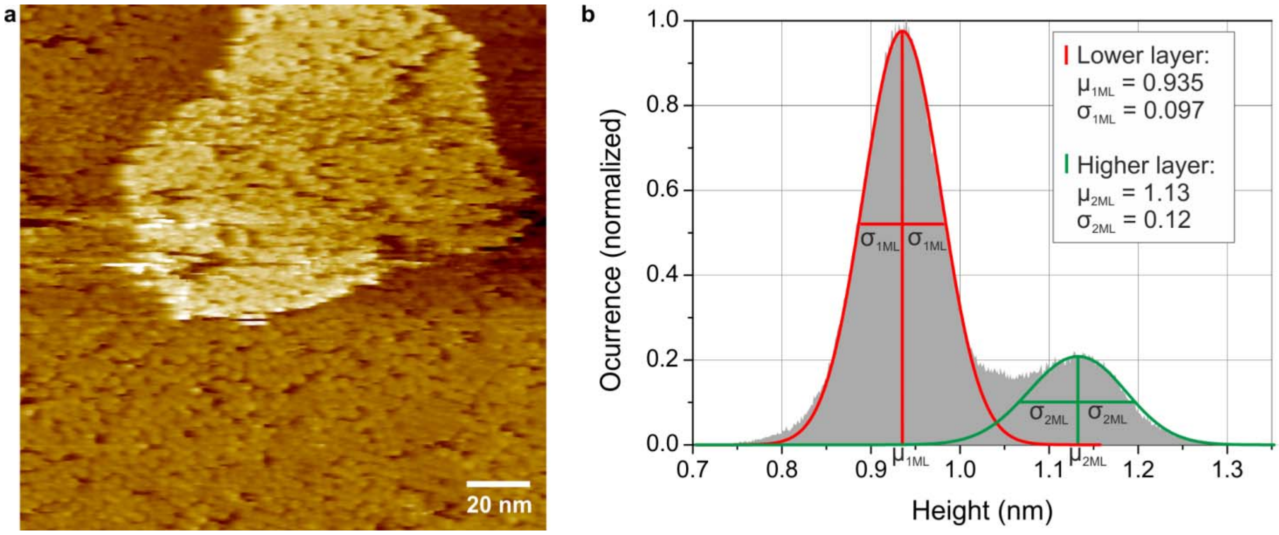

Supplementary Figure 8 | Island of templated bilayer of *E,E*-8-mer·C_{60}. (**a**) STM height image of a double layer from highly concentrated heptanoic acid solution (~10^{-4} M) of *E,E*-8-mer·C_{60} on TMA. (**b**) Histogram of the image in (**a**) with its mean values μ and standard errors σ.

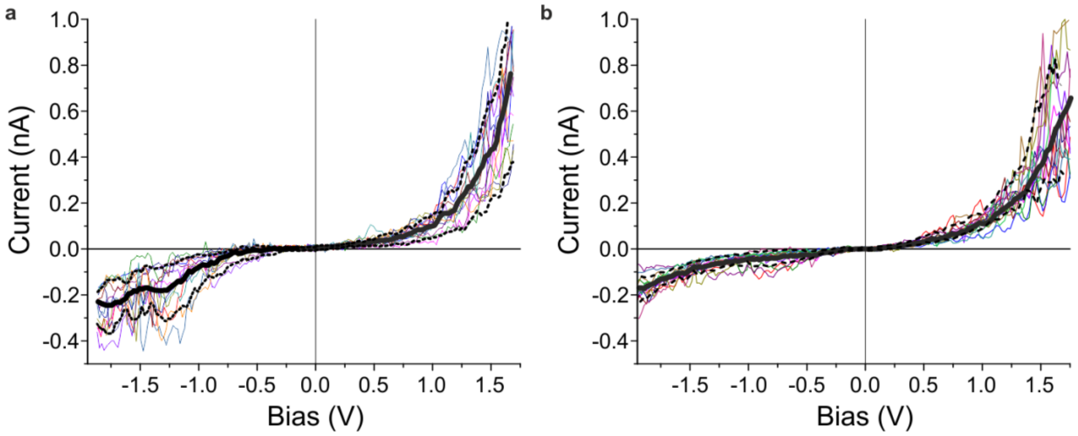

Supplementary Figure 9 | Scattering of averaged I-Vs in Fig. 4. 15 different I-V curves through (**a**) one monolayer and (**b**) two monolayers of complex *E*,*E*-8-mer·C_{60} (color lines) and the average value (thick black line). A dashed line connects the limits of the error bars for each graph.

Supplementary Note 1: Validation of force field parameters for *E,E*-8-mer

The potential energy function of the CGenff follows that of CHARMM[3], which consists of bonded and non-bonded terms, Supplementary Equation (1), where the force constants K for bond, angles and torsions; the equilibrium constants b_0, θ_0 ; the dihedral phase δ; the partial atomic charges q; and the Lennard–Jones parameters ε and σ need to be determined.

$$V_{\text{bonded}} = \sum_{\text{bonds}} K_b(b-b_0)^2 + \sum_{\text{angles}} K_\theta(\theta-\theta_0)^2 + \sum_{\text{dihedrals}} \sum_{n=1}^{n=6} K_{\phi,n}(1+\cos(n\phi-\delta))$$
$$V_{\text{nonbonded}} = \sum_{\text{charges}} \frac{q_i q_j}{4\pi\epsilon_0 r_{ij}} + \sum_{\text{LJ}} \epsilon_{ij}\left[\left(\frac{\sigma}{r_{ij}}\right)^{12} - 2\left(\frac{\sigma}{r_{ij}}\right)^{6}\right] \quad (1)$$

Molecule *E,E*-8-mer is a novel type of thiophene derivative macrocycle, therefore most of the force field parameters of Supplementary Equation (1) have to be calculated. An automatic assignment of the parameters could be done at paramchem.org[4,5], together with the correspondent penalty for using those parameters by analogy to other molecules. We did the fitting of the missing parameters using the force field toolkit[6] (fftk) implemented in VMD visualization software[7]. The molecule *E,E*-8-mer is divided into reduced molecule for ease of the calculation, especially those involved quantum mechanical calculations of torsional scans, Supplementary Fig. 10 shows the reduced molecules employed. Saturated and unsaturated aliphatic and aromatic hydrogens were automatically assigned the standard charges 0.09, 0.150 and 0.106. Similarly, the partial charges of saturated carbons in the groups -CH_3 and -CH_2 were assigned to the standard value 0.27 and 0.18, except for the carbon attached to thiophene units. Reduced compound I and II were used to determine the partial charges of the atoms labeled in Supplementary Fig. 10b. These charges were assigned by analogy to equivalent atoms in reduced compounds III and IV, which were then used to calculate the partial charges of the remaining atoms. Equivalent charges were assigned to chemically equivalent atoms of thiophene, ethylene and acethylene units; for example C3-C5 and C6-C7 in Supplementary Fig. 10.

Remaining bond, angle and dihedral parameters were fitted using reduced compounds III and IV. Supplementary Table 1 summarizes the CGenff force field parameter fitting using fftk, only those

parameters not assigned by analogy are reported. Lennard–Jones parameters for each atom type are assigned by analogy.

C_{60} molecules were assigned atom type CG2R61 (aromatic) and no partial charge.

To validate the assigned parameters we calculated the binding energy of the complex *E,E*-8-mer·C_{60} by computing the quantity $E_{\text{bind}} = E_3 - (E_2 + E_{\text{C60}})$, where E_2, E_{C60} and E_3 are the total energies after a 2000 steps minimization of the macrocycle *E,E*-8-mer, C_{60} and the complex *E,E*-8-mer·C_{60} respectively. The formation of the complex is favored by (10±5) kcal/mol, which is fair agreement with enthalpy of complexation estimated by van't Hoff plot from binding constant in toluene-d_8 solutions, –(5.5±0.4) kcal/mol[8].

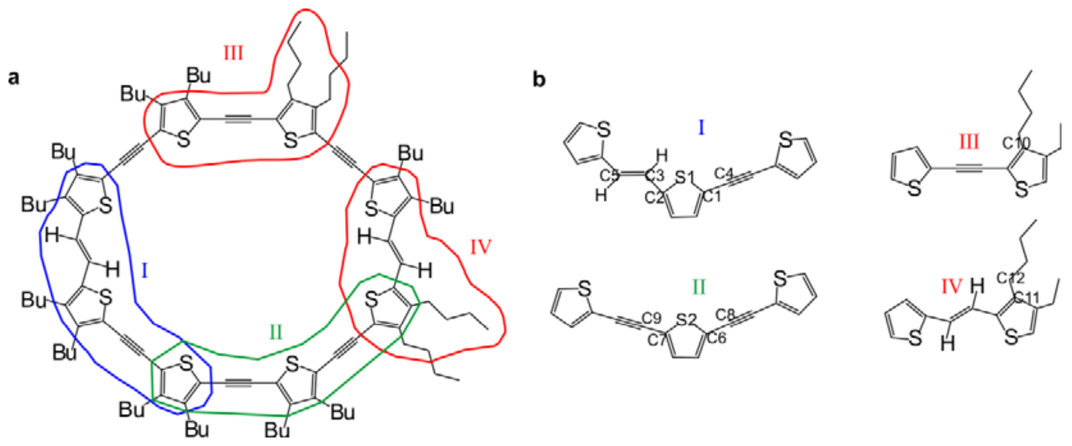

Supplementary Figure 10 | Reduced molecules used for the parametrization of molecule *E,E*-8-mer.

Supplementary Table 1 | Partial atomic charges of selected atoms in Supplementary Fig. 11 and CGenFF force field parameters using fftk. Force constants K_b, K_θ and $K\phi$ are given in kcal mol^{-1}, b_0 in Å, θ_0 and δ in degrees.

Partial atomic charges		Bonds			Angles			Dihedrals			
Atom	charge	Bond	K_b	b_0	Angle	K_θ	θ_0	Dihedral	$K\phi$	n	δ
S1	-0.099	CG2R51 CG1T1	354.50	1.405	SG2R50 CG2R51 CG1T1	86.557	121.91	CG2R51 CG2DC1 CG2DC1 HGA4	2.6010	2	180
C1	0.098	CG2R51 CG2DC1	375.79	1.457	CG2R51 CG2R51 CG1T1	81.719	127.62	CG2R51 CG2R51 CG2R51 CG2DC1	5.9920	2	180
C2	0.028				CG2R51 CG1T1 CG1T1	48.438	177.81	CG2R51 SG2R50 CG2R51 CG2DC1	27.555	1	0
C4	0.008				SG2R50 CG2R51 CGDC1	90.186	121.15	CG2R51 CG2R51 CG2DC1 HGA4	1.4370	2	180
C3 C5	-0.115				CG2R51 CG2R51 CGDC1	105.19	125.32	SG2R50 CG2R51 CG2DC1 HGA4	1.0530	2	0
S2	-0.100				CG2R51 CG2DC1 HGA4	62.113	117.03	SG2R50 CG2R51 CG2DC1 CG2DC1	2.2720<br>0.5620	2<br>4	180<br>0
C6 C7	0.098				CG2R51 CG2DC1 CG2DC1	88.719	125.37	CG2R51 CG2R51 CG2DC1 CGDC1	0.2660	2	0
C8 C9	0.008				CG2R51 CG321 CG321	58.35	114.00	CG2R51 CG2DC1 CG2DC1 CG2R51	26.256	1	0
C10	-0.121							CG2DC1 CG2R51 CG2R51 CG321	2.6060	2	180
C11	-0.100							CG2R51 CG2R51 CG2R51 CG1T1	6.5880	2	180
C12	-0.115							CG2R51 SG2R50 CG2R51 CG1T1	7.494	2	180
								CG321 CG2R51 CG2R51 SG2R50	20.846	1	0
								CG2R51 CG2R51 CG2R51 CG321	7.6410	2	180
								CG321 CG2R51 CG2R51 CG1T1	5.9940	1	180
								CG321 CG2R51 CG2R51 CG321	13.116	1	180

Supplementary Note 2: MD simulation of monolayer and bilayer of templated *E,E*-8-mer·C_{60} on graphene

A graphene slab was constructed to simulate the HOPG substrate as it has been shown to describe well the adsorption of polyaromatics onto this substrate[9].

One unit cell of templated complex *E,E*-8-mer·C_{60} was built up on top of the 24 TMA honeycomb network as shown in Supplementary Fig. 11a. The hexagonal unit cell has equal cell constants $a_3 = b_3 = \sqrt{3} \times a_{TMA}$ = 29.78 Å, making the (√3×√3)R30° superstructure. In the initial configuration, all the alkyl chains are parallel to the substrate plane. After running 2 ns of MD simulation at 300 K, the system adopts the configuration showed in Supplementary Fig. 11b. Analysis of the last 50 frames of the simulation (1 ns) showed that the alkyl chain are uneven distributed between back-folded upwards (41%), downwards (26%) and in-plane (33%).

Similarly, a bilayer of complex *E,E*-8-mer·C_{60} is built above the first monolayer as depicted in Supplementary Fig. 12a. After 2 ns of MD simulation at 300 K, the distribution of the alkyl chains changes to more butyl groups in-plane, 40% on the first ML and 41% on the second ML; back-folded upwards, 28% on first ML and 29% on second ML; and downwards, 33% and 30% for first and second ML respectively. The redistribution of the orientation of alkyl chains is due to steric effects by the proximity of the macrocycles of the two monolayers, 4.8 Å (see Fig. 5d in main text), while the mean distance between C_{60} molecules is ~7.1 Å.

Supplementary Fig. 13 show the evolution of the non-bonded terms of Supplementary Equation (1) for the MD simulations of 1ML and 2ML of complexes *E,E*-8-mer·C_{60} on TMA|graphene. The decrease of the energy upon formation of the second monolayer is obtained by the difference $E_{vdW2ML} + E_{Elect2ML} - (E_{vdW1ML} + E_{Elect1ML})$, which for the last nanosecond is –(169±30) kcal/mol.

Supplementary Fig. 14 shows a STM simulated MD trajectory image by averaging 100 snapshots taking every 20 ps (2 ns MD simulation). Each frame is rendered according to the contribution of each atom to the tunneling current[10], proportional to the expression $I_0 e^{-\beta z}$, where β is the current attenuation factor and z the height of the molecule/atom.

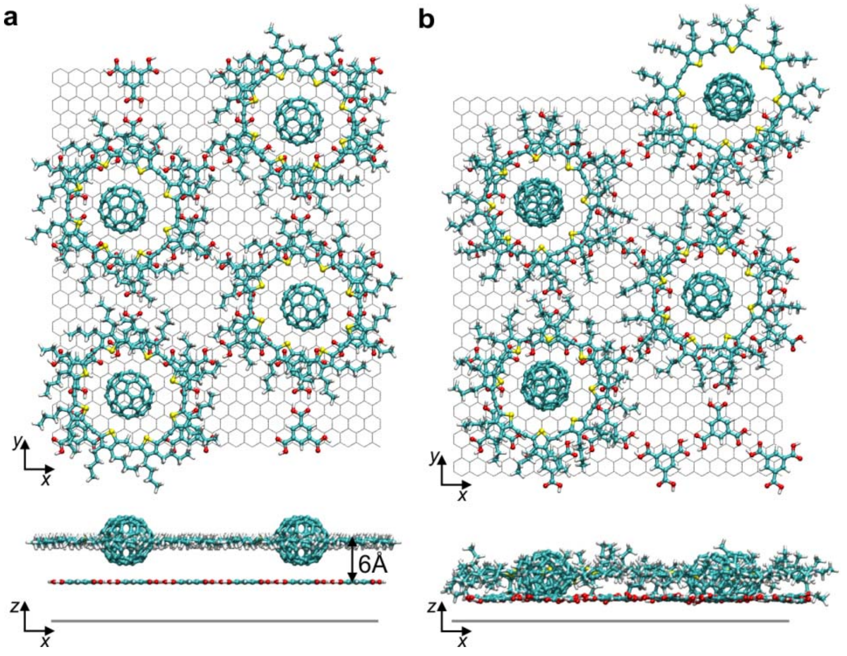

Supplementary Figure 11. a. Top and side view of the initial guess for a one monolayer of *E,E*-8-mer·C_{60} on top of a TMA network on a graphene slab. **b.** Top and side view after 2 ns of MD simulations at 300 K.

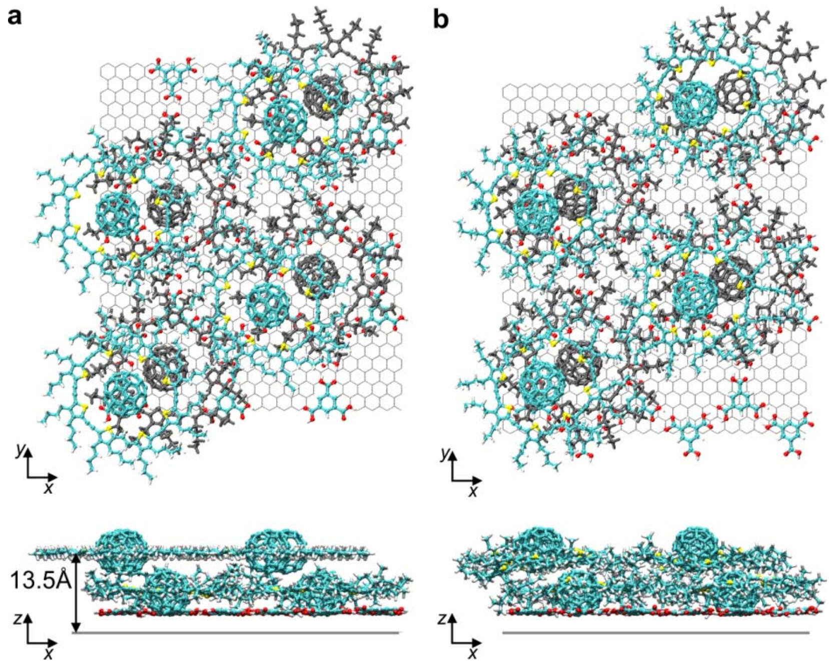

Supplementary Figure 12. a. Top and side view of the initial guess for two monolayers of *E,E*-8-mer·C_{60} on top of a TMA network on a graphene slab. **b.** Top and side view after 2 ns of MD simulations at 300 K.

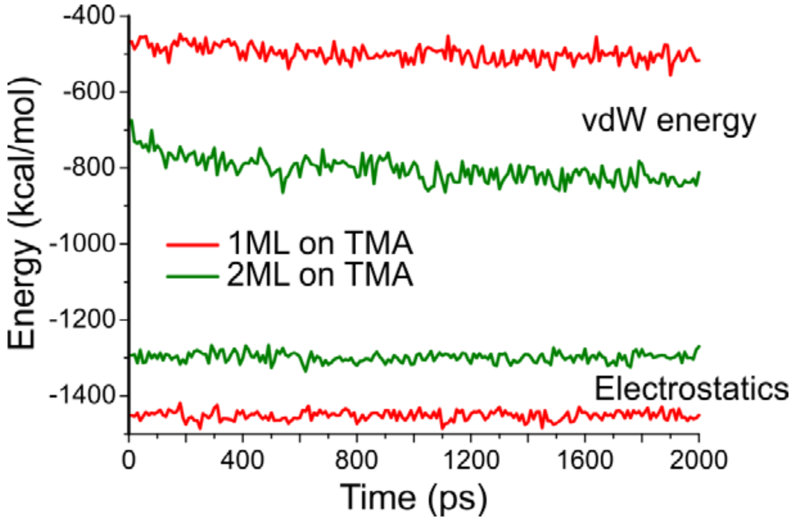

Supplementary Figure 13. Evolution of non-bonded potential terms for the MD simulations depicted in Supplementary Figs 11 and 12.

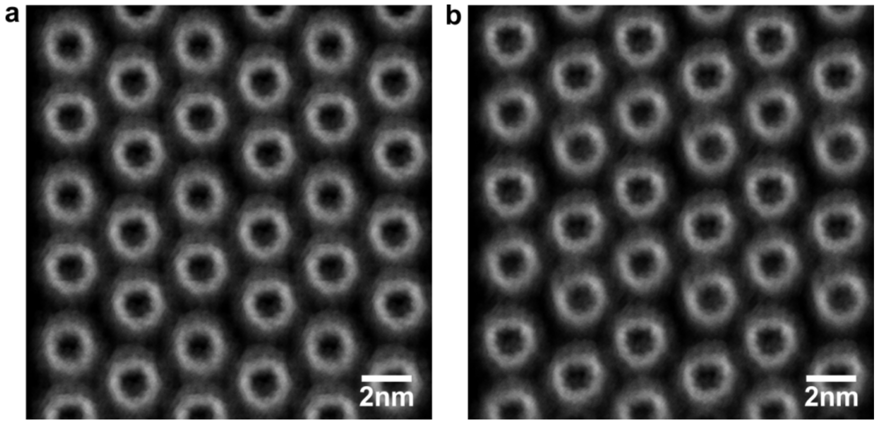

Supplementary Figure 14. STM simulated images averaged from 2 ns of MD simulation of two templated monolayers of *E,E*-8-mer·C_{60}**. a.** First monolayer, unit cell *a* = (3.1±0.8) nm, *b* = (3.1±0.9) nm and θ = (60.5±0.6)° **b.** Second monolayer, unit cell *a* = (3.1±0.8) nm, *b* = (3.1±0.8) nm and θ = (60.7±0.8)°

Supplementary Methods

Density functional theory (DFT) calculations

The molecular geometries and molecular orbitals of the molecules have been computed by using density functional theory (DFT). Becke three-parameter[11] hybrid exchange functional combined with the Lee–Yang–Parr[12] correlation functional (B3LYP) was used with the 6-31G++(d,p) basis set for geometry optimization. Gaussian09[13] program, running on an Debian 7 Linux server in 1 Intel Core i7-4770 processor at 3.40 GHz was used for calculations. Molecular frontier orbitals images were visualized with Gaussview5 software and isosurface here (Supplementary Fig. 4b) and in the main text (Fig. 2a) with VMD[7] software.

Supplementary References

1. Jäckel, F., Wang, Z., Watson, M. D., Müllen, K. & Rabe, J. P. Nanoscale array of inversely biased molecular rectifiers. *Chem. Phys. Lett.* **387,** 372–376 (2004).
2. Böhme, T., Simpson, C. D., Müllen, K. & Rabe, J. P. Current–Voltage Characteristics of a Homologous Series of Polycyclic Aromatic Hydrocarbons. *Chem. – Eur. J.* **13,** 7349–7357 (2007).
3. Brooks, B. R. *et al.* CHARMM: A program for macromolecular energy, minimization, and dynamics calculations. *J. Comput. Chem.* **4,** 187–217 (1983).
4. Vanommeslaeghe, K. & MacKerell, A. D. Automation of the CHARMM General Force Field (CGenFF) I: Bond Perception and Atom Typing. *J. Chem. Inf. Model.* **52,** 3144–3154 (2012).
5. Vanommeslaeghe, K., Raman, E. P. & MacKerell, A. D. Automation of the CHARMM General Force Field (CGenFF) II: Assignment of Bonded Parameters and Partial Atomic Charges. *J. Chem. Inf. Model.* **52,** 3155–3168 (2012).
6. Mayne, C. G., Saam, J., Schulten, K., Tajkhorshid, E. & Gumbart, J. C. Rapid parameterization of small molecules using the force field toolkit. *J. Comput. Chem.* **34,** 2757–2770 (2013).
7. Humphrey, W., Dalke, A. & Schulten, K. VMD: Visual molecular dynamics. *J. Mol. Graph.* **14,** 33–38 (1996).
8. Shimizu, H. *et al.* Synthesis, Structures, and Photophysical Properties of π-Expanded Oligothiophene 8-mers and Their Saturn-Like C_{60} Complexes. *J. Am. Chem. Soc.* **137,** 3877–3885 (2015).
9. Björk, J. *et al.* Adsorption of Aromatic and Anti-Aromatic Systems on Graphene through π−π Stacking. *J. Phys. Chem. Lett.* **1,** 3407–3412 (2010).
10. Palma, C.-A. *et al.* Two-dimensional soft supramolecular networks. *Chem. Commun.* **51,** 17297–17300 (2015).
11. Becke, A. D. Density-functional thermochemistry. III. The role of exact exchange. *J. Chem. Phys.* **98,** 5648–5652 (1993).

12. Lee, C., Yang, W. & Parr, R. G. Development of the Colle-Salvetti correlation-energy formula into a functional of the electron density. *Phys. Rev. B* **37,** 785–789 (1988).

13. Frisch, M. J. *et al. Gaussian 09 Revision D.01*.
